# Retraction Note: Long non-coding RNA HIF1A-AS2 facilitates adipose-derived stem cells (ASCs) osteogenic differentiation through miR-665/IL6 axis via PI3K/Akt signaling pathway

**DOI:** 10.1186/s13287-023-03355-y

**Published:** 2023-05-10

**Authors:** Ruoyu Wu, Jihao Ruan, Yongjin Sun, Mengyu Liu, Zhuang Sha, Cunyi Fan, Qingkai Wu

**Affiliations:** 1 Institute of Microsurgery on Extremities, Shanghai Jiao Tong University Affiliated Sixth People’s Hospital, Shanghai, 200233 China; 2 Department of Orthopaedics, Shanghai Jiao Tong University Affiliated Sixth People’s Hospital, No.600 Yishan Road, Xuhui District, Shanghai, 200233 China; 3 Department of Obstetrics and Gynecology, Shanghai Jiao Tong University Affiliated Sixth People’s Hospital, Shanghai, 200233 China; 4 Institute of Nervous System Diseases, Xuzhou Medical University, Xuzhou, 221004 Jiangsu China

**Retraction Note to: Stem Cell Research & Therapy (2018) 9:348** 10.1186/s13287-018-1082-z

The Editors-in-Chief have retracted this article. After publication, concerns were raised regarding the binding site between IL-6 mRNA and miR-665. Specifically, the sequence of IL6 3'UTR reported in this article appears to refer to ILR6 rather than IL6. Additionally, in the TargetScan database, miR-665 does not appear to be among the predicted miRNAs targeting IL6. Further checks by the Publisher identified the following concerns with the presented data:

In Figs. 4f and 6g, three images appear to originate from the same sample (4f ALP Vector and sh-NC, and 6g Antagomir-665+sh-IL6).

In Figs. 5f, 6f and 7a, all western blot images have highly similar background features, and a number of bands appear to have straight vertical edges, which are inconsistent with the backgrounds.

The authors have stated that Figs. 4f and 6g indeed contain errors, and that the western blots are not presented in their original state. They have provided raw data to address these concerns; however, these data contain further inconsistencies and highly similar images. The Editors-in-Chief therefore no longer have confidence in the data and conclusions of this article.

Ruoyu Wu has not explicitly stated whether they agree to this retraction notice. Jihao Ruan, Yongjin Sun, Mengyu Liu, Zhuang Sha, Cunyi Fan and Qingkai Wu have not responded to any correspondence from the editor or publisher about this retraction.

